# Kinetics Study of Al Extraction from Desilicated Coal Fly Ash by NaOH at Atmospheric Pressure

**DOI:** 10.3390/ma14247700

**Published:** 2021-12-13

**Authors:** Andrei Shoppert, Irina Loginova, Dmitry Valeev

**Affiliations:** 1 Department of Non-Ferrous Metals Metallurgy, Ural Federal University, 620002 Yekaterinburg, Russia; loginova_irina@mail.ru; 2 Laboratory of Sorption Methods, Vernadsky Institute of Geochemistry and Analytical Chemistry of the Russian Academy of Sciences, 119991 Moscow, Russia; dmvaleev@yandex.ru

**Keywords:** mullite, coal fly ash, alkaline, leaching, neural network, machine learning, kinetics, shrinking core model

## Abstract

The most promising source of alumina in the 21st century is the coal fly ash (CFA) waste of coal-fired thermal plants. The methods of alumina extraction from CFA are often based on the pressure alkaline or acid leaching or preliminary roasting with different additives followed by water leaching. The efficiency of the alumina extraction from CFA under atmospheric pressure leaching is low due to the high content of acid-insoluble alumina phase mullite (3Al_2_O_3_·2SiO_2_). This research for the first time shows the possibility of mullite leaching under atmospheric pressure after preliminary desilication using high liquid to solid ratios (L:S ratio) and Na_2_O concentration. The analysis of the desilicated CFA (DCFA) chemical and phase composition before and after leaching has been carried out by inductively coupled plasma optical emission spectrometry (ICP-OES) and X-ray diffraction (XRD). The morphology and elemental composition of solid product particles has been carried out by scanning electron microscopy with energy-dispersive X-ray spectroscopy (SEM-EDX). An automated neural network and a shrinking core model (SCM) were used to evaluate experimental data. The Al extraction efficiency from DCFA has been more than 84% at T = 120 °C, leaching time 60 min, the L/S ratio > 20, and concentration of Na_2_O—400 g L^−1^. The kinetics analysis by SCM has shown that the surface chemical reaction controls the leaching process rate at T < 110 °C, and, at T > 110 °C after 15 min of leaching, the process is limited by diffusion through the product layer, which can be represented by titanium compounds. According to the SEM-EDX analysis of the solid residue, the magnetite spheres and mullite acicular particles were the main phases that remained after NaOH leaching. The spheric agglomerates of mullite particles with non-porous surface have also been found.

## 1. Introduction

Two types of ash are produced during coal combustion in boilers: coal bottom ash (CBA) and collected in the waste gas system coal fly ash (CFA), which together are called the coal ash (CA). The chemical and phase composition of the CA depends on many factors, including the coal deposit, coal combustion methods and parameters, etc. [1]. There are two types of ash that are obtained by different combustion methods [2]: in pulverized coal boilers and using a circulating fluidized bed. CA from the fluidized bed boilers is formed at a lower temperature (850–950 °C), does not have microsphere particles, and the main phase of alumina is amorphous glassy mass. CA from the pulverized coal boilers formed during the melting of coal mineral inclusions of coal at T = 1200–1500 °C. Thus, the main alumina phase in such CA is mullite represented by spherical particles.

CFA contains many valuable components, primarily alumina and silica. The alumina content in some types of ash can be comparable to bauxite; this ash is called high alumina fly ash (HAFA). The Inner Mongolia Autonomous Region (China) provides a special type of coal; after the combustion process, it generates ash with a high alumina content, up to 50% [3]. HAFA is a promising raw material for alumina production [4] due to the limited reserves of bauxite located in China [5]. However, alumina content in the typical ash is about 25–30% [6], while silica is more than 50%.

Many hydrometallurgical methods of alumina extraction from CFA have been developed to date. They can be divided into alkaline [7,8,9], acidic [10,11,12,13], and roasting (combined pyro- and hydrometallurgical) [14,15,16,17,18,19,20], when the roasting product is leached by water or mineral acids.

Roasting methods are based on the conversion of aluminum from insoluble minerals into water-soluble ones during its reaction with different additives (NaOH [21], Na_2_CO_3_ [22], NaCl [23], CaCO_3_ [24], CaCl_2_ [25] at T = 400–1200 °C). Aluminum can be extracted from CFA after roasting by acid leaching at atmospheric pressure [17,26]. The roasting flux dissolves into the solution and cannot be reused. So, these methods require higher energy and reagents consumption and are less environmentally friendly compared to hydrometallurgical methods.

Acidic methods allow a selective Al extraction from CFA by HCl [23,27], H_2_SO_4_ [28], and NH_4_HSO_4_ [29] using high-pressure reactors. However, CFA leaching at atmospheric pressure leads to a low Al extraction degree [4]. It was found that the process can be intensified by hydrofluoric acid (HF) addition [30] or using a combination of acid and alkaline leaching, but equipment with high corrosion resistance is required.

Alkaline methods used for the Al extraction from bauxite are less efficient for CFA [31]. This is due to the fact that CFA contains a high amount of amorphous glassy silica, which dissolves faster than Al-containing minerals [32]. The presence of SiO_3_^2−^ in solution simultaneously with NaAlO_2_^−^ leads to the precipitation of desilication product (DSP, Na_6_[Al_6_Si_6_O_24_] Na_2_X (where X is different inorganic anions) [33]. A large amount of Al and Na are lost with the DSP. The silica content in the CFA can be reduced by the preliminary alkali desilication. Simultaneously, the alumina content in the solid residue increases from 20–30% to 30–50%. After the CFA alkali desilication process, the pure solutions of sodium silicate (Na_2_SiO_3_) are obtained. Silica solution after CFA desilication is used for mesoporous silica nanoparticles or various types of zeolites production [5,34,35,36,37,38,39].

However, using the conventional desilication method at the low L:S ratio, the Si extraction at the preliminary alkaline desilication stage does not exceed 60%; as much as ≈12–14 wt. % Na_2_O was found in the solid residue after desilication [40]. This is because a part of alumina in the CFA is contained in an amorphous glassy mass that readily dissolves and leads to DSP precipitation. To reduce the alkali losses during the desilication process, Aphane M. et al. suggested leaching of a readily soluble Al from the glass mass by acid [41].

Further Al extraction from desilicated CFA is carried out in high-pressure reactors [42], since most of the Al is contained in the CFA in refractory mullite (3Al_2_O_3_·2SiO_2_). The low Al extraction degree from mullite can be also related to solid films of amorphous glassy mass and DSP on the CFA surface [4]. Using hydrothermal conditions at T > 250 °C in the presence of a certain amount of lime, it is possible to extract more than 90% of Al by obtaining DSP with a low Al and Na content [43]. Therefore, the DSP formation is an important issue for Al extraction from CFA by alkaline methods, and the development of new methods for Si and Al extraction from CFA by NaOH is required.

Our previous study showed that it is possible to completely remove the amorphous glassy mass from the CFA without the DSP formation during alkaline desilication at the high liquid to solid (L/S) ratio and Na_2_O concentration [32]. This fact is associated with the retention of silica in the metastable area [44,45] that helps to extract more than 90% of Si with minimal alkali losses. At the high leaching duration and temperature (>110 °C), it was found that, in addition to amorphous glassy mass, mullite begin to dissolve at the atmospheric pressure. The purpose of this study is to evaluate the Al extraction by a concentrated NaOH solution at atmospheric pressure from the desilicated by the new method CFA. The kinetics and mechanism of the mullite leaching have been studied.

## 2. Materials and Methods

### 2.1. Materials and Reagents

DCFA obtained by the desilication of the coal fly ash from the Reftinskaya thermal power plant in Asbest, Russia (GPS coordinates: 57.112213, 61.704545) was used as a raw material. The desilication process was carried out at T = 120 °C, L:S = 20, leaching time 20 min, and 400 g L^−1^ of Na_2_O [32]. Caustic alkali (JSC Soda, Sterlitamak, Russia) and aluminum hydroxide (Al(OH)_3_) (JSC “BaselCement-Pikalevo”, Pikalevo, Russia) of the analytical grade were used in the present research. The alkaline solutions were prepared by dissolving a predetermined amount of the solid NaOH in 300 mL of distilled water. After complete dissolution, the volume was adjusted by water to obtain a solution with the Na_2_O concentration of 330, 360, or 400 g L^−1^ (C_Na2O_). To study the effect of the Al concentration in the solution on the leaching process, solutions of various initial concentrations-190 and 380 g L^−1^ Al_2_O_3_ (C_Al2O3_^0^) were prepared by dissolving the Al(OH)_3_ in a hot alkaline solution.

### 2.2. Analysis

The mineral composition of the solid samples was determined by X-ray diffraction (XRD) using a Difrei-401 diffractometer (JSC Scientific Instruments, Saint Petersburg, Russia) using a Cr-Kα radiator with 2θ angles ranging from 15° to 140°. The operating mode of the X-ray source was 25 kW/4 mA with 30 min of exposure time. Match 3 software was used to process the diffraction data. The quantitative analysis of crystalline phases in the DCFA sample was carried out by the Rietveld quantitative phase analysis (RQPA) method, using “FullProf” and “Match! 3” software (Crystal impact, Bonn, Germany) for analysis.

Chemical analysis was performed after complete dissolution of the solid residue by a mixture of concentrated hydrofluoric, sulfuric, and nitric acids; the residue was subsequently fused with soda and boric acid at 950 °C and leached using 1 N HCl solution, which was followed by inductively coupled plasma optical emission spectrometry (ICP-OES) analysis, using a spectrometer Vista Pro (Varian Optical Spectroscopy Instr., Mulgrave, Australia). For quality assurance, samples were analyzed twice. The carbon contents analysis was performed via a fractional gas analyzer CS-600 (LECO Corporation, St. Joseph, MI, USA). The loss on ignition (LOI) was determined by calcination at 1000 °C for 60 min.

The morphological forms and the elemental composition of the main minerals of the samples were determined by means of scanning electron microscopy with energy-dispersive X-ray spectroscopy (SEM-EDX, Vega III, Tescan, Brno, Czech Republic). In order to reduce the charge formed on the surface, a current-conducting coating was applied to the surface of the samples via a Q150R ES coater (Quorum Technologies, UK). The coating was applied by cathode sputtering; the materials of the coating were gold (to determine the spatial location of the particles) and carbon (to determine the structure of the samples and perform X-ray microanalysis.

The particle size distribution and mean particle size analysis were performed by the laser diffraction method (LD) using an Analysette 22 NanoTec (Fritsch, Idar-Oberstein, Germany). The specific surface area of the samples was determined via the Brunauer–Emmett–Teller method (BET) using NOVA 1200e (Quantachrome Instruments, Boynton Beach, FL, USA). Before BET analysis, all samples were subjected to degassing under vacuum at 200 °C for 12 h.

### 2.3. Experiments

**Figure 1:**
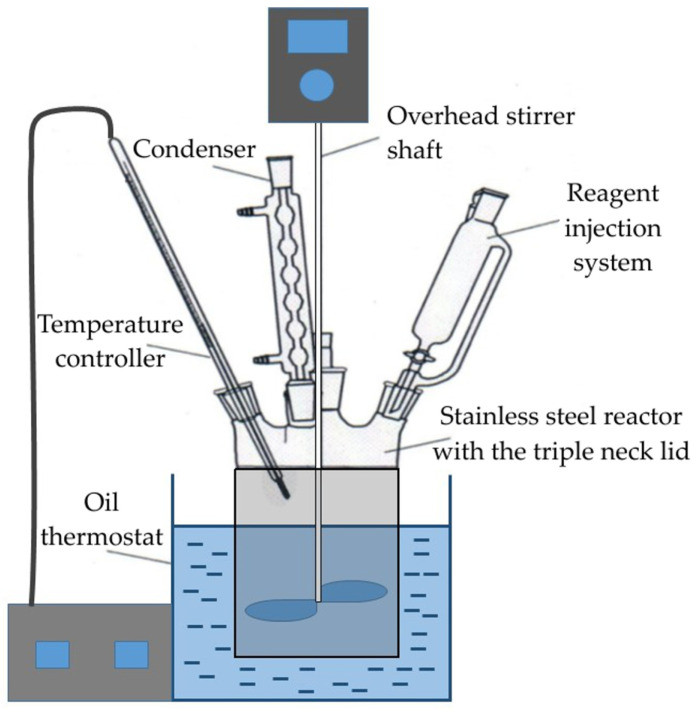
Schematic of the NaOH leaching experimental set-up.

Preliminary desilication of CFA and DCFA leaching by NaOH was carried out in the thermostated 0.5 L stainless steel reactor (Figure 1). The reactor has openings for injecting chemical reagents as well as for temperature control and the recycling of evaporated water through a water-cooled condenser. The stirring speed in all experiments was 400 rpm: previously [32], it was found that leaching efficiency does not improve at a higher rotation speed. The DCFA was added to the solution with the Na_2_O concentration of 330, 360, or 400 g L^−1^ and an initial concentration of Al_2_O_3_ 0, 190, and 380 g L^−1^. After leaching, the pulp was filtered; the solid residue was dried at 110 °C for 240 min before analysis using ICP-OES.

### 2.4. Experimental Data Evaluation

The extraction degree of Al and Si from DCFA after NaOH leaching was calculated by Equation (1):

α = [(m_1_ × Me_1_)/(m_2_ × Me_2_)] × 100%, (1)

where Me_1_ is the Al or Si content in the solid residue obtained after DCFA leaching by NaOH, %; m_1_ is the weight of the solid residue; Me_2_ is the content of the Al or Si in the DCFA, %; m_2_ is the weight of the DCFA load in the experiment, g.

Statistical-based automated neural network (SANN) was used for modeling of DCFA leaching by NaOH. SANN is an artificial intelligent method that adjusts the result of modelling until the desired quality is obtained. “STATISTICA 13” software was used for SANN modelling via a multilayer perceptron (MLP) method. MLP implies the creation of a neural network consisting of input, hidden, and output layers, where hidden and output layers are the activation function that is executed progressively to obtain an output value depending on the input parameters. The input parameters were the leaching duration (τ, min), the L:S ratio (L:S), the temperature (T, °C), Na_2_O concentration (C_Na2O_, g L^−1^), initial Al_2_O_3_ concentration (C_Al2O3_^0^, g L^−1^), and the initial mean particle size (r^0^, μm). The output layer consisted of one response variable: extraction of Al (wt. %). MLP was set to a minimum of 3 hidden layers and a maximum of 10 hidden layers. The number of networks to train was 50, and the networks to save was 5. Other parameters were automated by the software. The SANN modeling process implies that the matrix structure is not needed.

The kinetic parameters and the coefficients of determination were calculated using “non-linear curve fit analysis” in commercial software, which is based on the non-linear least-squares method. This method reduces the number of calculations and figures. The main advantage of this method is the possibility to evaluate the quality of fitting experimental data by the non-linear chi-square test (χ2) [46]. The different SCM models were manually added as an “explicit function”. “Independent variable” was the time of leaching, “Dependent variable” was the fraction of reacted solid or the degree of conversion; “parameters” was the apparent rate constant.

## 3. Results and Discussion

### 3.1. Characterization of the Raw CFA and DCFA

**Figure 2:**
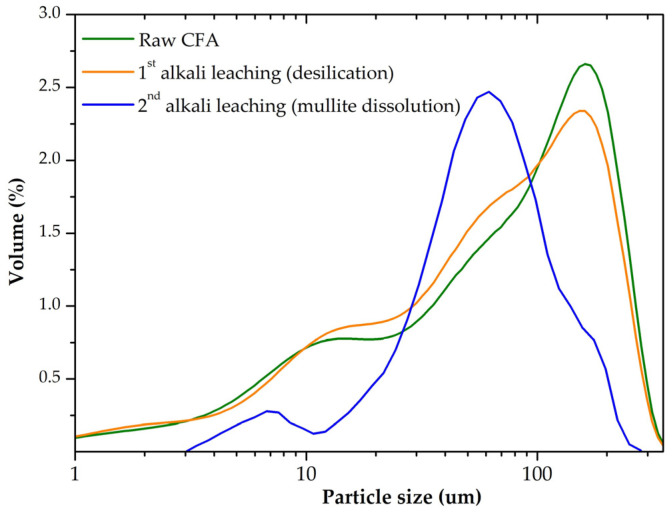
The particle size distribution of the raw coal fly ash (CFA, green curve), desilicated CFA (DCFA, orange curve), and DCFA after NaOH leaching at T = 120 °C, L:S ratio = 20, τ = 60 min (blue curve).

The raw CFA was desilicated at the parameters that exclude high losses of Al due to mullite dissolution (leaching time < 20 min); i.e., it is suggested that during the desilication stage, only amorphous glassy mass was extracted. The yield of DCFA was 40.5 wt. % of the raw CFA sample mass. The Al and Si extraction at the desilication stage were 17.3 and 80.7 wt. %, respectively. The particle size distribution of the CFA, DCFA, and the solid residue after mullite leaching is shown in Figure 2. DCFA used in the kinetic study was subjected to a sieve analysis to obtain three fractions: −50 µm, +50–71 µm, and +71 µm. The average particle size of each fraction was: 48 µm, 62 µm, and 87 µm. The chemical composition of these three fractions and the raw CFA is shown in Table 1.

**Figure 3:**
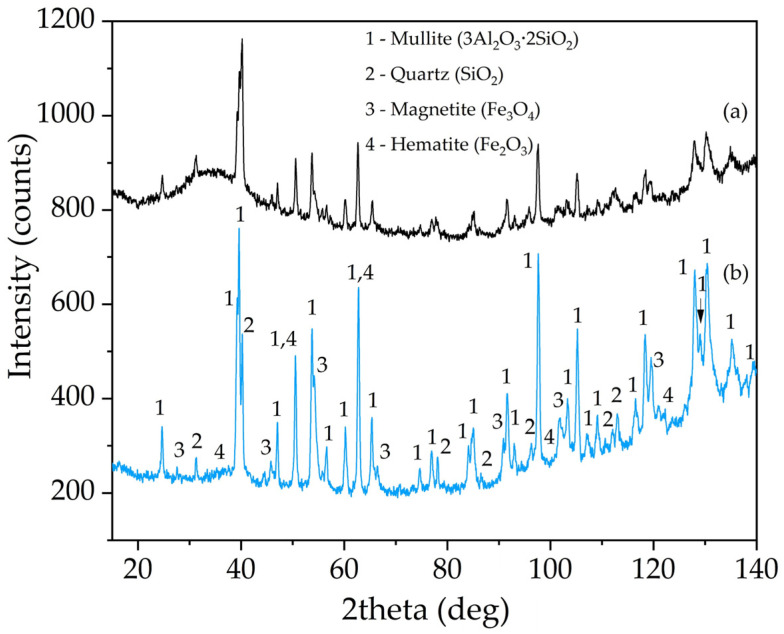
XRD pattern of the CFA from Reftinskaya TPP, Asbest, Russia (**a**) and DCFA (**b**).

Figure 3 shows the XRD pattern of the raw CFA and DCFA. The DCFA mainly consists of three mineral phases: mullite, magnetite (Fe_3_O_4_), and quartz (SiO_2_). A glassy amorphous phase (from 20° to 50° 2Theta) the raw CFA was eliminated by alkali leaching at the desilication stage. Therefore, the remaining 82.7% of Al and 19.3% of Si from the raw CFA are mainly contained in mullite and quartz, which was confirmed by the Rietveld method. The quantitative analysis of crystalline phases in the DCFA sample is shown in Table 2. According to Table 2, more than 78% of DCFA is represented by mullite. However, it should be noted that unburned coal and other aluminosilicates are X-ray amorphous.

**Figure 4:**
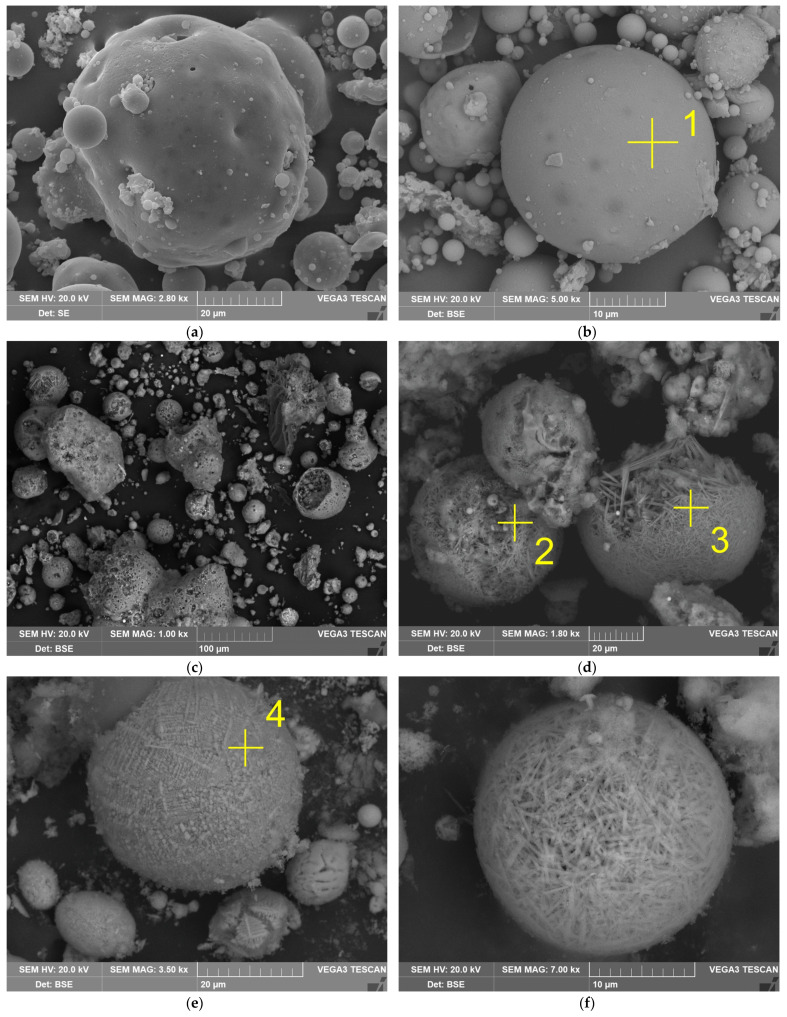
The SEM images of CFA at magnitude 2800 (**a**); mullite covered by the amorphous glassy phase at magnitude 5000 (**b**); DCFA at magnitude 1000 (**c**); DCFA at magnitude 1800 (**d**); magnetite particles at magnitude 3500 (**e**); spherical agglomerate of mullite particles at magnitude 7000 (**f**).

The effect of the raw CFA desilication on the morphology and the chemical composition of the particles was evaluated using the SEM-EDX analysis (Figure 4, Table 3). The SEM-EDX images in Figure 4 demonstrate that the raw CFA mullite was represented by the spheres with a smooth surface. After desilication, the porosity of the particles was greatly increased, which was confirmed by the BET analysis in our previous study [32]. The specific surface area of the raw CFA is 0.81 m^2^ g^−1^, whereas the specific surface area of the DCFA–15.70 m^2^ g^−1^. Figure 4d shows that mullite acicular particles remain after the amorphous glassy phase dissolution from the surface. The agglomerate of the acicular mullite particles is spherical, as it was in the raw CFA. Magnetite is also represented by spheres (Figure 4c). The EDX analysis of magnetite and mullite particles is shown in Table 3. The high porosity of the DCFA and exposure of the acicular mullite particles explains the increase in its reactivity. Therefore, the subsequent alkali or acid mullite leaching can be accomplished under atmospheric pressure.

### 3.2. The Effect of Leaching Parameters on the Mullite Dissolution

In this research, the mullite atmospheric leaching from DCFA by highly concentrated alkaline solutions was investigated using SANN and SCM. The chemical reaction of the interaction of mullite with caustic alkali can be represented by Equation (2).

3Al_2_O_3_·2SiO_2 (s)_ + 10NaOH _(aq)_ + 7H_2_O _(l)_ = 6NaAl(OH)_4 (aq)_ + 2Na_2_SiO_3 (aq)_. (2)

As was revealed in our previous study [32], the use of high alkaline concentrations and L:S ratios exclude the DSP formation via retention of Si in the metastable area. This allows the complete extraction of alumina from fly ash despite how much silica was in the raw CFA. Moreover, the boiling point of highly concentrated NaOH solution is higher than 120 °C [47]. This allows us to use temperatures above 100 °C without high-pressure equipment. The matrix of experiments and the results of Al and Si extraction degree are shown in Table 4.

**Figure 5:**
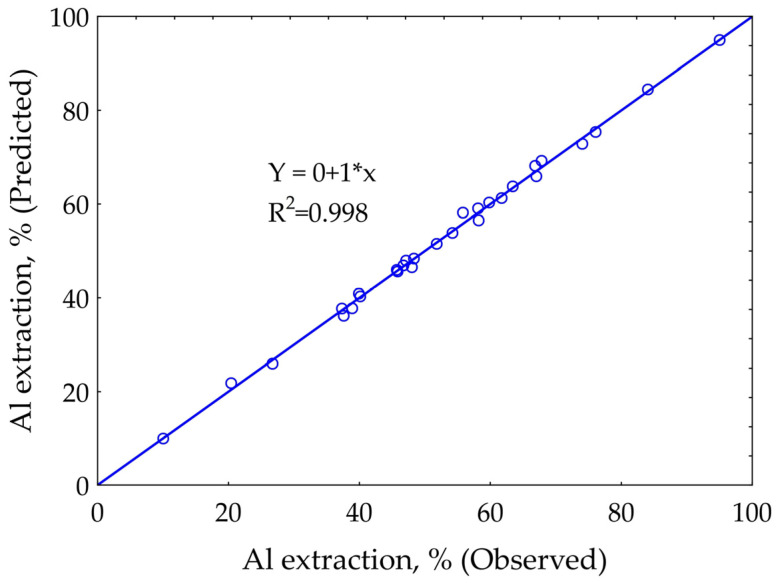
Coefficient of determination for SANN-based alumina extraction from DCFA model.

As was shown by Xie et al. and Shokri [42,43], using machine learning allows us to get more accurate models than using mathematical methods. The best fit SANN model obtained for the extraction of alumina is a multilayer perceptron (MLP) 6.9.1, where six is the number of input parameters, nine is the number of hidden layers, and one is the number of output layers. Experimental data and values predicted using the resulting network are in good agreement (R^2^ = 0.988), Figure 5.

**Figure 6:**
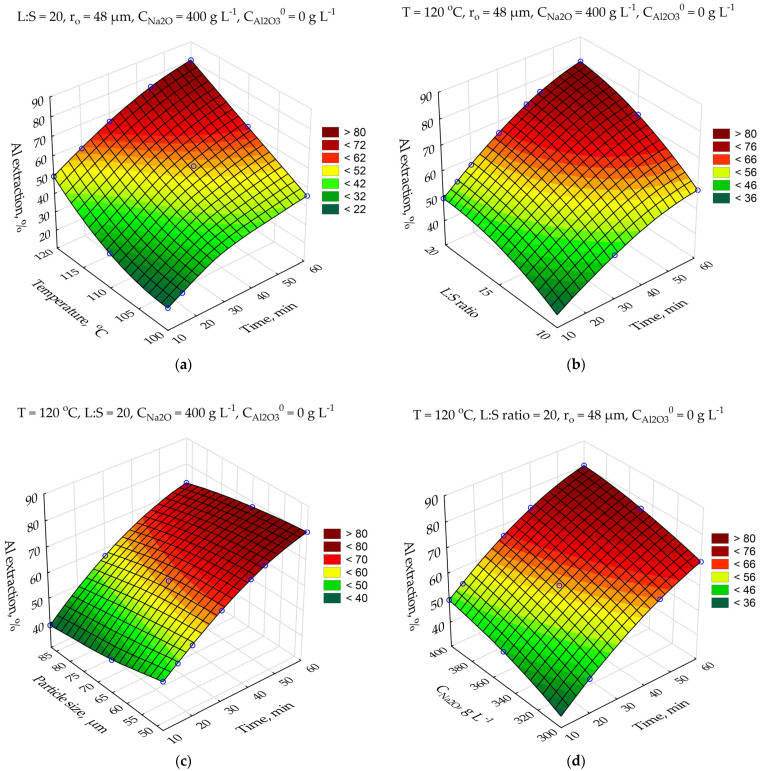

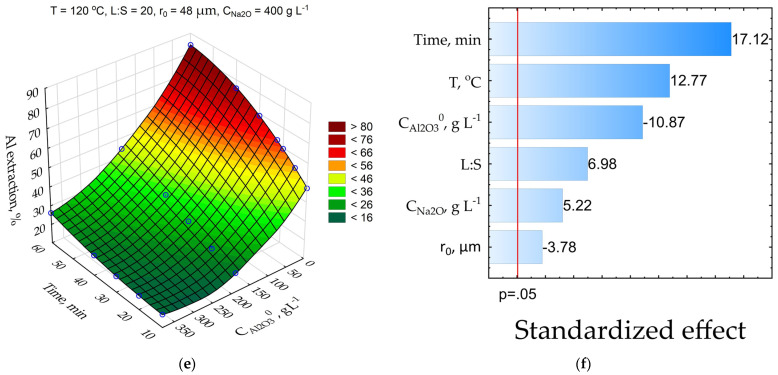
Neural network response surfaces for effect of time and temperature on the Al extraction (**a**); effect of time and L:S ratio on the Al extraction (**b**); effect of time and particle size on the Al extraction (**c**); effect of time and Na_2_O concentration on the Al extraction (**d**); effect of time and initial Al_2_O_3_ concentration on the Al extraction (**e**); Pareto chart (**f**). Blue points are the experimental data.

The response surfaces predicted by the SANN for Al extraction degree depending on the leaching duration (τ, min), the L:S ratio (L:S), the temperature (T, °C), Na_2_O concentration (C_Na2O_, g L^−1^), initial Al_2_O_3_ concentration (C_Al2O3_^0^, g L^−1^), and the initial mean particle size (r^0^, μm) are shown on Figure 6.

The major effect (Figure 6) on Al extraction degree is caused by leaching time, temperature, the Al concentration in the solution, and the L:S ratio. Increasing the temperature from 100 to 120 °C allows us to increase the Al extraction degree after 60 min from 46 to 84%. This may indicate that the surface chemical reaction is the limiting stage of the process. An increase in the initial concentration of Al_2_O_3_ in the solution from 0 to 380 g L^−1^ leads to a decrease in Al extraction degree from 84 to 51%. This is connected to the fact that the solution is already sufficiently saturated with aluminum, and approaching the equilibrium concentration can lead to a reverse precipitation reaction. In this situation, external diffusion could be the limiting stage. The effect of the average particle size and the Na_2_O concentration is significantly lower, which is more common for the kinetic limiting stage. This observation is also confirmed by the results presented in the Pareto chart (Figure 6f). The kinetic studies were conducted to understand which stage is limiting the leaching process.

### 3.3. Kinetic Study

The SANN model obtained on the basis of the experimental data from Table 4 was used to study kinetics of the leaching process with help of various shrinking core models [48]. These models imply that during the leaching of particles, their core shrinks to the center, leaving behind a layer of inert product. In this case, substances insoluble in alkali can serve as an inert product as well as refractory compounds that require increased pressure to leach.

Three models of the shrinking core were used in this work. Equation (3) can be used to describe a process limited by a surface chemical reaction:

[1 − (1 − X)^1/3^] = k_1_t, (3)

where X is the degree of conversion; k_i_ is the apparent rate constant of Equation (3); t is the leaching time, min.

When the leaching rate is limited by the diffusion through inert product layer, the kinetic Equation (4) can be used:

[1 − 2/3X − (1 − X)^2/3^] = k_2_t. (4)

where k_2_ is the apparent rate constant of Equation (4).

If the leaching rate is limited by the diffusion through the liquid film, then Equation (5) can be used:

X = k_3_t. (5)

where k_3_ is the apparent rate constant of Equation (5).

The non-linear least squares method was used to fit the obtained data into the equations of the shrinking core model.

**Figure 7:**
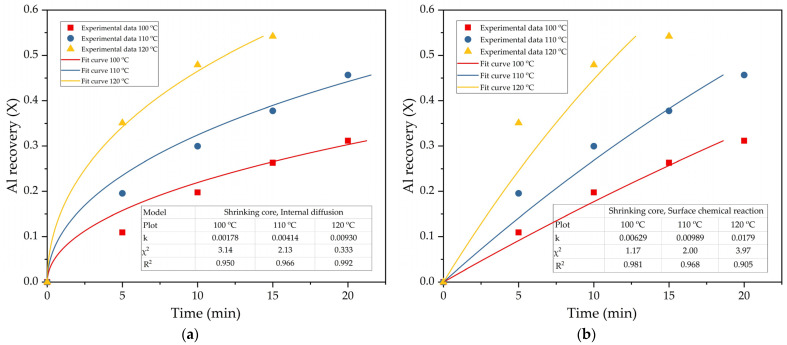
The results of fitting obtained data (points) to Equation (2) and Equation (3) (lines): the diffusion through the product layer (**a**); the surface chemical reaction (**b**) for the effect of temperature.

**Figure 8:**
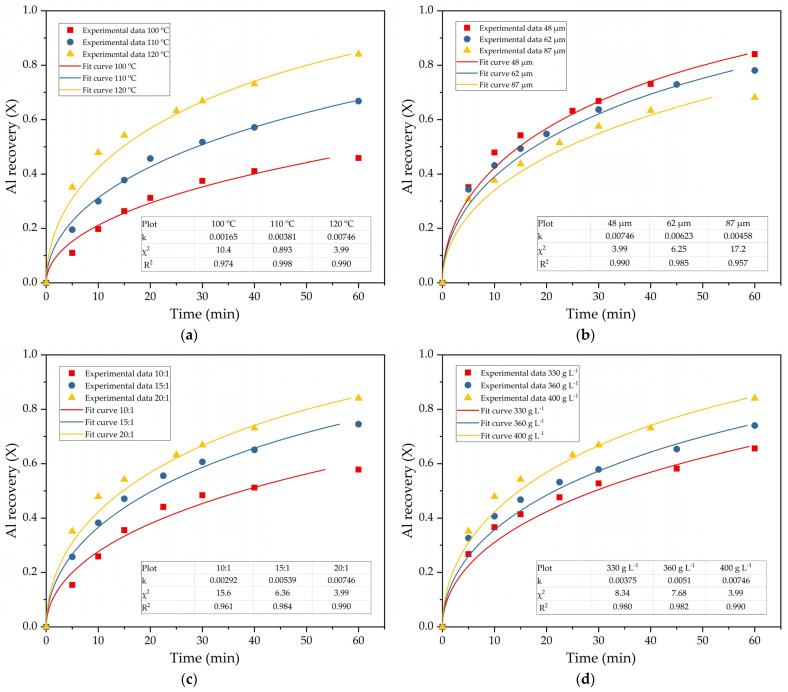

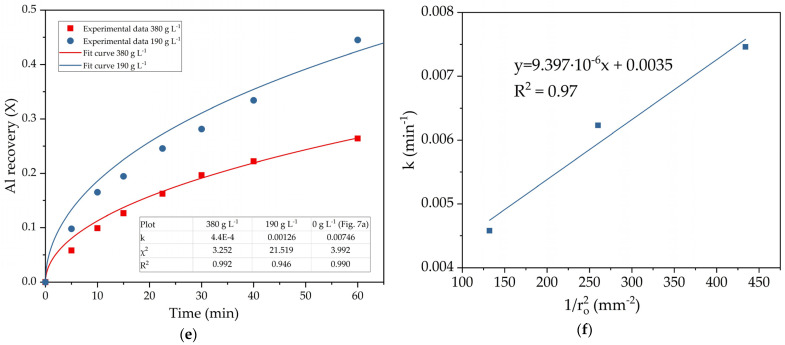
The results of fitting obtained data (points) to Equation (3) (lines): the diffusion through the product layer for the effect of temperature (**a**); the diffusion through the product layer for the effect of particle size (**b**); the diffusion through the product layer for the effect of L:S ratio (**c**); the diffusion through the product layer for the effect of C_Na2O_ (**d**); the diffusion through the product layer for the effect of C_Al2O3_^0^ (**e**) and relation between the apparent rate constant and the reverse square of particle radius (**f**).

Equations (3) and (4) are best suited to describing the leaching process of mullite (Figure 7). The data presented on Figure 7a,b show that at temperatures below 110 °C and leaching time less than 20 min, the surface chemical reaction shrinking core model provides the best fit to the experimental data. While at temperatures above 110 °C and leaching time of more than 20 min (Figure 8a), the data are more suitable for the modeling of diffusion through the product layer. Therefore, Equation (3) was chosen to fit data obtained by varying other parameters (Figure 8). The fixed parameters, if not stated otherwise, were as follows: T = 120 °C, L:S = 20, C_Na2O_ = 400 g L^−1^, r_0_ = 48 µm, C_Al2O3_^0^ = 0 g L^−1^.

Leaching efficiency at T = 100 °C is much lower than at T = 120 °C; this explains why the shrinking core model for surface chemical reaction is more suitable at low temperature (Figure 7b). At high temperatures and leaching time, a layer of inert product becomes thicker. Therefore, the leaching rate can be limited by the diffusion of the alkaline solution through the product layer. The nature of the product layer appearing during mullite leaching in case of Al_2_O_3_ and SiO_2_ simultaneous extraction requires further research using SEM-EDX analysis.

An increase in the average particle size only slightly reduces the leaching efficiency (Figure 8b). The low effect of particle size can relate to the high porosity of DCAF. However, according to Gok et al. [49], if diffusion through the product layer controls the reaction rate, there should be a linear relation between the apparent rate constant (k_2_) and the reverse square of particle radius (1/r_0_^2^). A dependence between k_2_ obtained in Figure 8b and 1/r_0_^2^ values is shown in Figure 8f. Linear relation with R^2^ = 0.97 confirms that diffusion through the product layer is the rate-limiting step for this process.

The high effect of solution concentration and L:S ratio (Figure 8c–e) indicates that the amount of free alkaline in the solution is essential for the leaching process, since DSP begins to form at a low L:S ratio and high initial alumina concentration [41].

**Figure 9:**
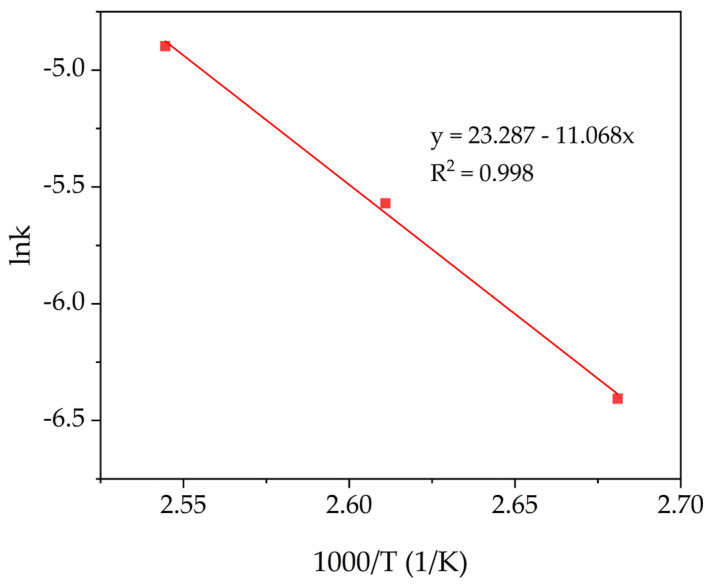
Relationship between lnk from Figure 7a and 1000/T.

The apparent activation energy (E_a_) was calculated using the values of k_2_ obtained in Figure 8a (constant rates at different temperatures). The linear fit shown in Figure 9 was used to determine the E_a_ according to the Arrhenius Equation (6):

k = k_0_ exp (−E_a_/RT). (6)

where k_0_ is the pre-exponential factor; E_a_ is the apparent activation energy, kJ/mol; R is the universal gas constant, 8.314 J/mol·K; and T is the reaction temperature, K.

According to the slope obtained in Figure 9, the E_a_ value is 92.0 kJ/mol. Therefore, it implies that the rate-limiting step of leaching of mullite, especially at low temperatures, is the chemical reaction. This is because the leaching of refractory mullite requires high activation energy even after the dissolution of glassy amorphous mass from the surface of the particles. Thus, according to SCM and the E_a_ value, the process of mullite dissolution is limited by the surface chemical reaction at low temperatures and leaching time and by diffusion through the product layer at the 120 °C and later stages of leaching. To reveal the nature of a product layer that inhibits the leaching process, solid residue characterization was performed.

### 3.4. Solid Residue Characterization

The chemical composition of the solid residue obtained at T = 120 °C, L:S ratio = 20, τ = 60 min, C_Na2O_ = 400 g L^−1^, and C_Al2O3_^0^ = 0 g L^−1^ is presented in Table 5. The yield of solid residue was 33.95% of the initial DCFA sample mass. It could be seen that iron and carbon content have increased significantly in the residue contrary to the initial DCFA. A high amount of silica and alumina still can be observed, which points out that not all mullite was extracted after 60 min of leaching. However, the extraction degree of Si (Table 4) and Al (on DCFA mass basis) at these parameters were 88.2 and 84.0 wt. %, respectively. On the raw CFA mass basis, Si and Al extraction degree at the mullite leaching stage were 17.0 and 66.7 wt. %, respectively. Thus, the Si and Al extraction degree from the raw CFA after two leaching stages were 80.7 + 17.0 = 97.7% and 17.3 + 66.7 = 84.0%, respectively. At the same time, Na_2_O content was still very low; it means that DSP was not formed during the leaching of DCFA at such an L:S ratio and Na_2_O concentration.

**Figure 10:**
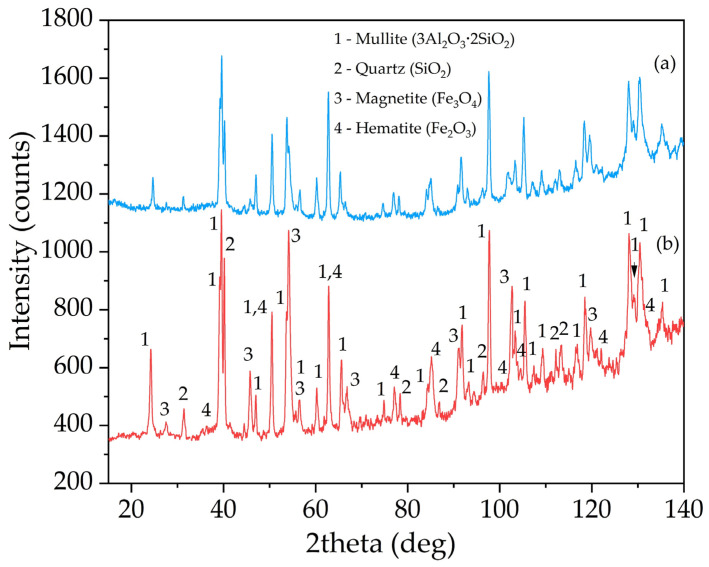
XRD pattern of the DCFA (**a**) the solid residue obtained after mullite leaching from DCFA at T = 120 °C, L:S ratio = 20, τ = 60 min, C_Na2O_ = 400 g L^−1^, C_Al2O3_^0^ = 0 g L^−1^ (**b**).

**Figure 11:**
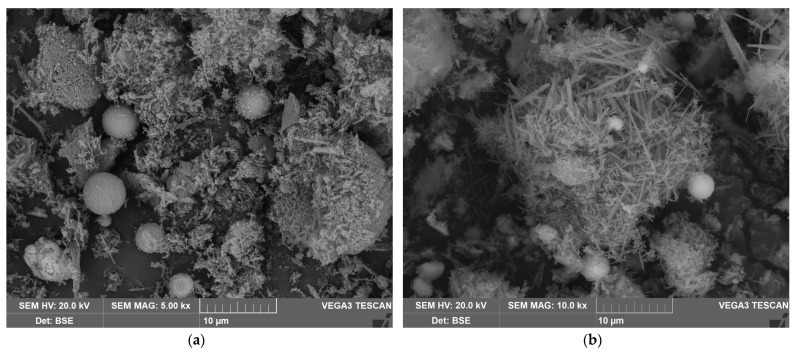

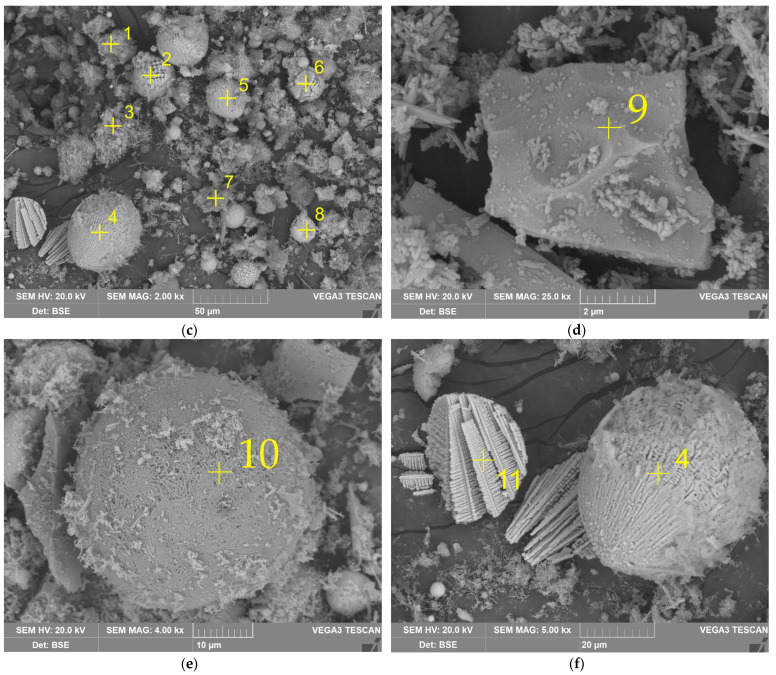
The SEM images of the solid residue at 5000 magnitude (**a**) and at 10,000 magnitude (**b**); the SEM images with the EDX analysis at 2000 magnitude (**c**); the SEM images with the EDX analysis of quartz particle at 25,000 magnitude (**d**); mullite particle at 4000 magnitude (**e**) and magnetite particles at 5000 magnitude (**f**) (yellow crosses indicate places of SEM-EDX analysis; the elemental composition is shown in Table 6).

The X-ray diffraction pattern of the solid residue after mullite leaching from DCFA is shown in Figure 10. The morphology and elemental composition of the solid residue particles were investigated by SEM-EDX (Figure 11 and Table 6). The surface area and porosity of the solid residue were studied by the BET method (Table 7).

As can be seen in Figure 10, the mullite peaks have not changed, while the magnetite peaks have increased significantly. Peaks of quartz were also increased in comparison with mullite. This fact suggests that only mullite is predominantly leached out, while the other phases remain unleached. The presence of the mullite peaks indicates that 60 min of leaching at 120 °C and a high concentration of Na_2_O is not sufficient to dissolve minerals as refractory as mullite. However, according to chemical analysis and the yield of the residue, more than 80% of mullite was leached out, as well as quartz.

The data obtained above are confirmed by the SEM-EDX (Figure 11 and Table 6). Figure 11a,b show that the spherical agglomerates of mullite particles are destroyed during the leaching process and the single acicular particles are seen on the surface. The EDX analysis has been done to clarify the chemical composition of the particles (Table 6). According to the analysis, there are still spherical particles of magnetite (Figure 11f), non-porous mullite aggregates (Figure 11e), and nonuniform particles of quartz (Figure 11c).

**Figure 12:**
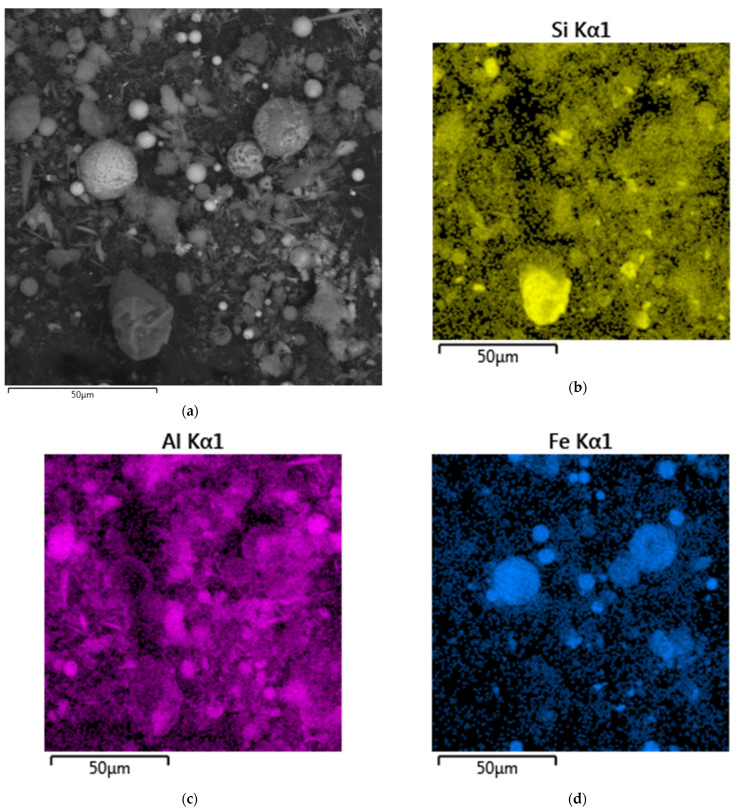

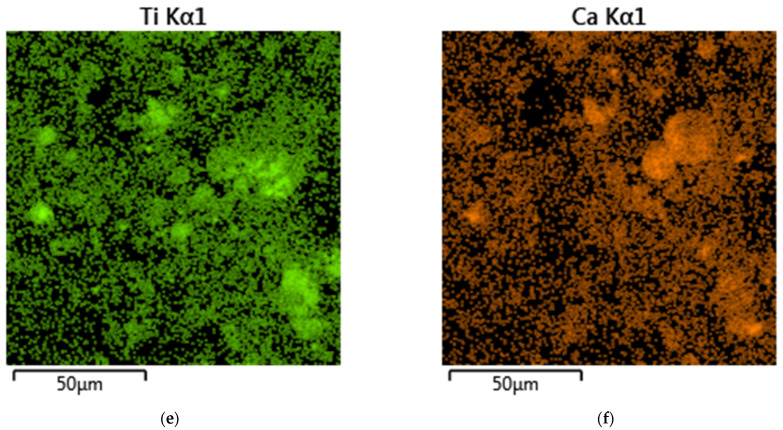
Mapping of DCFA surface using SEM-EDX analysis: BSE image of the surface (**a**); mapping of the Si distribution (**b**); mapping of the Al distribution (**c**); mapping of the Fe distribution (**d**); mapping of the Ti distribution (**e**); mapping of the Ca distribution (**f**).

Except for Al, Si, and Fe, the solid residue contains a high amount of Ca and Ti (Table 5), the phases of which are not seen on the XRD pattern (Figure 10). The correlation of Ca and Ti with other minor elements was evaluated by SEM-EDX mapping of the surfaces of the particles, as can be seen in Figure 12. Ti was found to be concentrated on the surface of the Al-rich phase, i.e., mullite. Ti mapping is also partially correlated with Ca-rich phases. The association of Ti with the Fe-rich phase, on the contrary, is low. Thus, it can be assumed that Ti is partially dissolved; then, it is precipitated on the mullite surfaces in the form of insoluble Na and Ca-containing compounds, as it has a place when diasporic bauxites are leached with highly concentrated alkaline solutions [50]. These Ti compounds can serve as the product layer that inhibits intraparticle diffusion. Therefore, the addition of lime or Fe(II) ions [50] is needed to reduce the Ti inhibition effect. However, mullite spheres (Figure 10e) with low porosity surface remain unleached even after three stages of alkaline leaching (not described in this article), and they have no Ti on the surface. It is possible that the dense packing of mullite particles in these agglomerates reduces their reactivity. Again, the high activation energy confirms that the surface chemical reaction could be the rate-limiting stage of the process. Therefore, high-pressure leaching is necessary for complete Al extraction from CFA. On the other hand, an increase in temperature will also increase the precipitation rate of DSP, which will lead to large losses of aluminum and alkali.

According to the data in Table 7, the DCFA specific surface area does not change after NaOH leaching. This means that in this case, there is no porous reaction product, and the dissolving of mullite particles leaves the magnetite particles with the same particle size and porosity.

**Figure 13:**
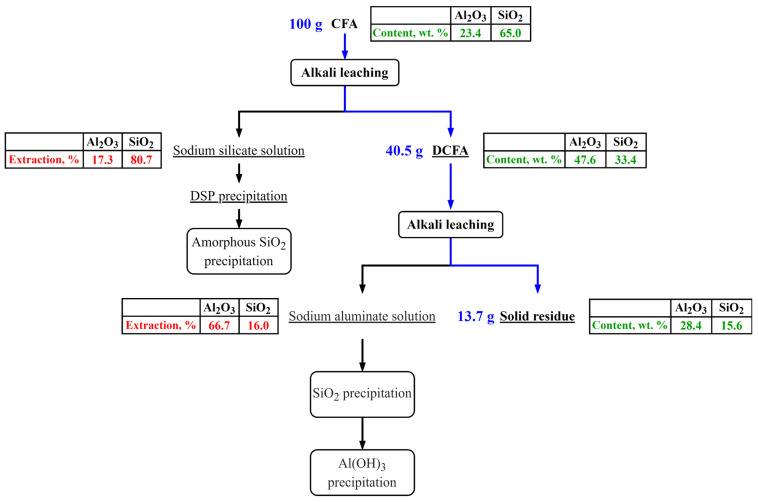
The schematic flow chart of the CFA alkaline atmospheric leaching after preliminary desilication and the extraction efficiency of Al and Si.

Figure 13 shows the schematic flow chart of the CFA alkaline atmospheric leaching after preliminary desilication and the extraction efficiency of Al and Si on each stage of the process at optimal parameters. As it was shown above, at the desilication stage about 17 wt. % of Al was dissolved. To enhance the Al extraction degree from the CFA, the DSP can be precipitated from the solution obtained at the desilication stage by addition of the DSP seed and stirring 60–120 min at 100–200 °C. The Al from the DSP can be further extracted by sintering it with soda followed by water or acid leaching [34]. The Si that was extracted at the mullite leaching step can be separated from Al by the stepwise precipitation that will be discussed in our future research.

## 4. Conclusions

This article showed that the atmospheric pressure alkaline leaching of mullite from preliminary desilicated CFA is possible at the optimized parameters. Using the artificial neural network method and shrinking core model, it was established that the leaching time, temperature, and initial concentration of alumina are essential to dissolve more than 80% of mullite. The main conclusions are as follows:

1. To extract mullite at atmospheric pressure, preliminary desilication at a high L:S ratio is necessary in order to accept DSP formation and expose the surface of mullite particles.
2. According to the response surfaces obtained by the SANN method, at T = 120 °C, L:S ratio = 20, τ = 60 min, C_Na2O_ = 400 g L^−1^, and C_Al2O3_^0^ = 0 g L^−1^, the Al extraction degree is 84%. A very low extraction degree is observed at the same parameters but C_Al2O3_^0^ = 390 g L^−1^. It indicates low solubility of mullite at a given temperature.
3. The kinetics analysis by a shrinking core model (SCM) has showed that the surface chemical reaction controls the leaching process rate at T < 110 °C, and, at T > 110 °C after 15 min of leaching, the process is limited by diffusion through the product layer, which can be represented by titanium compounds. The apparent E_a_ was 92.0 kJ/mol.
4. The unleached mullite in the solid residue is represented by individual acicular particles, as well as agglomerates with high alumina content and low porosity surface. The whole extraction efficiency of Si and Al after desilication and mullite leaching was more than 97% and 84%, respectively.

## Figures and Tables

**Table 1:** Chemical composition of the raw CFA and DCFA three size fractions: −50 µm, +50–74 µm, and +74 µm.

Size Fraction	Main Components, wt. %									
	SiO_2_	Al_2_O_3_	CaO	Fe_2_O_3_	TiO_2_	MgO	Na_2_O	K_2_O	LOI	C
Raw CFA	62.94	23.98	1.59	3.45	1.11	0.43	0.72	0.93	3.99	1.60
−50 µm	33.44	47.58	2.98	8.65	1.96	0.59	0.63	0.15	4.01	3.29
+50–71 µm	33.51	47.70	2.97	8.45	1.85	0.57	0.62	0.17	4.10	3.75
+71 µm	34.51	46.10	2.97	6.39	1.50	0.46	1.18	0.18	6.51	5.05

**Table 2:** Semi-quantitative analysis of mineral phases in DCFA.

Phase	Content %
Mullite	78.4
Quartz	10.7
Magnetite	7.3
Hematite	3.6
Total	100

**Table 3:** The result of EDX analysis of the raw CFA and DCFA (see Figure 4 for the spectra numbers).

Spectrum	O	Si	Al	Ca	Fe	Ti	Mg	Phase
1	48.9	28.7	19.8	0.3	1.2	-	0.5	Mullite covered by A-S ^1^
2	37.3	23.3	37.7	-	-	1.0	-	Mullite
3	42.3	17.1	40.6	-	-	-	-	Mullite
4	28.4	2.2	6.6	1.7	56.7	2.0	1.2	Magnetite

^1^—amorphous glassy mass.

**Table 4:** The matrix for planning experiments and the results of Al and Si extraction degree.

Time (min)	Temperature (°C)	L:S Ratio (mL/g)	r_0_ (μm)	C_Na_2_O_ (g L^−1^)	C_Al_2_O_3__^0^ (g L^−1^)	Al Extraction (%)	Si Extraction (%)
10	120	20	48	400	0	48.00	60.40
30	120	20	48	400	0	66.82	78.11
40	120	20	48	400	0	76.07	83.77
60	120	20	48	400	0	84.04	88.22
30	120	10	48	400	0	48.30	60.00
22.5	120	15	48	400	0	54.20	66.00
40	110	20	48	400	0	55.80	66.59
60	110	20	48	400	0	67.00	76.00
15	100	20	48	400	0	26.70	33.85
60	100	20	48	400	0	45.79	60.40
45	120	20	48	330	0	59.40	66.59
10	120	20	48	330	0	38.90	51.20
20	120	20	48	330	0	46.70	59.43
60	120	20	48	330	0	66.10	76.51
10	120	20	48	360	0	39.90	53.40
30	120	20	48	360	0	59.81	74.10
60	120	20	48	360	0	74.04	82.51
10	120	15	48	400	0	39.90	53.10
60	120	15	48	400	0	74.04	81.05
10	120	10	48	400	0	28.50	35.10
60	120	10	48	400	0	58.10	73.20
10	110	20	48	400	0	28.63	35.20
10	100	20	48	400	0	20.40	30.40
30	100	20	48	400	0	37.60	52.30
60	100	20	48	400	0	45.70	58.67
10	120	20	87	400	0	37.30	49.12
30	120	20	87	400	0	58.20	73.60
60	120	20	87	400	0	67.80	81.30
30	120	20	65	400	0	63.40	77.50
30	120	20	65	400	190	28.13	46.87
30	120	20	65	400	380	19.62	37.18
10	120	20	65	400	190	16.50	35.05
40	120	20	65	400	190	33.40	49.80
60	120	20	65	400	190	44.52	50.20
10	120	20	65	400	380	9.91	29.70
40	120	20	65	400	380	22.24	39.51
60	120	20	65	400	380	26.40	44.87

**Table 5:** Chemical composition of the solid residue after DCFA leaching by NaOH at T = 120 °C, L:S ratio = 20, τ = 60 min, C_Na2O_ = 400 g L^−1^, C_Al2O3_^0^ = 0 g L^−1^.

Main Components, wt. %									
SiO_2_	Al_2_O_3_	CaO	Fe_2_O_3_	TiO_2_	MgO	Na_2_O	K_2_O	LOI	C
15.56	28.4	6.28	26.28	8.60	0.53	0.1	0.01	11.86	10.67

**Table 6:** The result of EDX analysis of solid residue (see Figure 11 for the spectra numbers).

Spectrum	O	Si	Al	Ca	Fe	Ti	Mg	C	Phase
1	28.4	2.2	6.6	1.7	56.7	2.0	1.1	-	Magnetite + Mullite
2	18.3	0.4	1.9	-	77.0	0.4	0.9	-	Magnetite
3	57.4	10.0	28.8	0.7	1.9	1.3	-	-	Mullite
4	22.1	-	3.8	-	70.8	-	2.2	-	Magnetite
5	20.9	-	2.8	-	72.9	-	2.0	-	Magnetite
6	18.5	0.4	2.5	0.4	74.6	-	2.6	-	Magnetite
7	21.7	2.0	6.0	0.8	2.8	1.3	-	65.1	C
8	43.0	5.2	18.7	1.4	27.2	0.7	3.2	-	Magnetite + Mullite
9	44.8	34.0	13.9	1.3	3.5	2.5	-	-	Mullite + Quartz
10	45.7	11.5	42.3	-	-	-	-	-	Mullite
11	35.6	-	5.5	-	54.4	-	3.9	-	Magnetite

**Table 7:** The textural properties and particle size of the DCFA (size fraction +50–71 µm) and the solid residue after NaOH leaching at T = 120 °C, L:S ratio = 20, τ = 60 min.

Product	Specific Surface Area (BET) (m^2^ g^−1^)	Total Pore Volume (cm^3^ g^−1^)	Pore Diameter (nm)
DCFA	15.70	25	37.6
Solid residue	16.28	33	33.6

## Data Availability

The study did not report any data.
